# From feces to data: A metabarcoding method for analyzing consumed and available prey in a bird‐insect food web

**DOI:** 10.1002/ece3.4787

**Published:** 2018-12-21

**Authors:** Seppo Rytkönen, Eero J. Vesterinen, Coen Westerduin, Tiina Leviäkangas, Emma Vatka, Marko Mutanen, Panu Välimäki, Markku Hukkanen, Marko Suokas, Markku Orell

**Affiliations:** ^1^ Department of Ecology and Genetics University of Oulu Oulu Finland; ^2^ Biodiversity Unit University of Turku Turku Finland; ^3^ Spatial Foodweb Ecology Group University of Helsinki Helsinki Finland; ^4^ Ecological Genetics Research Unit University of Helsinki Helsinki Finland

**Keywords:** dietary ecology, DNA barcoding, fecal DNA, frass, insectivorous birds, Lepidoptera, metagenomics

## Abstract

Diets play a key role in understanding trophic interactions. Knowing the actual structure of food webs contributes greatly to our understanding of biodiversity and ecosystem functioning. The research of prey preferences of different predators requires knowledge not only of the prey consumed, but also of what is available. In this study, we applied DNA metabarcoding to analyze the diet of 4 bird species (willow tits *Poecile montanus*, Siberian tits *Poecile cinctus*, great tits *Parus major* and blue tits *Cyanistes caeruleus*) by using the feces of nestlings. The availability of their assumed prey (Lepidoptera) was determined from feces of larvae (frass) collected from the main foraging habitat, birch (*Betula* spp.) canopy. We identified 53 prey species from the nestling feces, of which 11 (21%) were also detected from the frass samples (eight lepidopterans). Approximately 80% of identified prey species in the nestling feces represented lepidopterans, which is in line with the earlier studies on the parids' diet. A subsequent laboratory experiment showed a threshold for fecal sample size and the barcoding success, suggesting that the smallest frass samples do not contain enough larval DNA to be detected by high‐throughput sequencing. To summarize, we apply metabarcoding for the first time in a combined approach to identify available prey (through frass) and consumed prey (via nestling feces), expanding the scope and precision for future dietary studies on insectivorous birds.

## 1 INTRODUCTION

Having knowledge of species' diets is the key to understanding trophic interactions in nature. Investigating the intricacies of food webs and variation within them for different years or areas contributes greatly to our understanding of biodiversity and ecosystem functioning (e.g., Paine, 1966, Petchey, Beckerman, Riede, & Warren, 2008, Yu et al., 2012). However, many ecological studies on predator–prey interactions have been carried out without knowledge of the exact composition of prey species in the diets. Identification of prey species using traditional methods like direct observations, video recordings or fecal microscopy presents various obstacles and shortcomings (e.g., Moreby & Stoate, 2000). This is especially true in insectivorous predators, whose prey is variable, small‐sized and easily disintegrates in the guts preventing direct identification (e.g., Clare, Fraser, Braid, Fenton, & Hebert, 2009; Clare et al., 2014, Kaunisto, Roslin, Sääksjärvi, & Vesterinen, 2017). Typically, this means that identification remains at a generalized prey categorization, such as “insect (herbivore) caterpillar” (functional grouping) or “Lepidoptera” (higher taxonomic grouping) (e.g., Naef‐Daenzer, Naef‐Daenzer, & Nager, 2000).

Recent methodological advances enable reliable and high‐resolution diet analyses even in insectivorous predators. Molecular dietary analysis—sequencing prey DNA from predator feces or gut contents and identifying them using a reference library (Clare et al., 2009; Eitzinger et al., 2018; Symondson, 2002; Vesterinen et al., 2016; Zaidi, Jaal, Hawkes, Hemingway, & Symondson, 1999)—can be used to reveal the exact food webs in ecosystems. The high‐throughput sequencing (HTS) approaches enable identification of species by simultaneously sequencing specimens of prey taxa in a bulk mixture (Gibson et al., 2014; Hajibabaei et al., 2006; King, Read, Traugott, & Symondson, 2008; Meusnier et al., 2008; Pompanon et al., 2012), making the diet analyses fast and cost‐effective. Certainly, the use of DNA metabarcoding has the potential to revolutionize ecological studies (e.g., Jedlicka, Sharma, & Almeida, 2012). For example, the temporal match/mismatch hypothesis (Cushing, 1969, 1990; Tiusanen, Hebert, Schmidt, & Roslin, 2016), fundamental to many studies of selection pressures on phenologies in trophic interactions between insectivorous birds and their prey (e.g., Visser, Noordwijk, Tinbergen, & Lessells, 1998), loses its conceptual basis if the actual species that interact are not known.

In this study, we test the metabarcoding methods in describing a boreal food web between insectivorous birds (four species of parids) and their invertebrate prey. According to previous studies (based on morphology, direct observations, etc.), breeding parids in northern Europe forage mainly on moth caterpillars feeding on birch leaves (Rytkönen, Koivula, & Orell, 1996; Rytkönen & Krams, 2003). Here, we will trial techniques to identify the diet for several bird species in the same food web in much greater detail than was previously possible. In addition, the availability of potential invertebrate prey in the birds' habitat is also monitored using the same methods. The prey consumed by the predators can be determined from prey DNA in the predators' feces (e.g., Vesterinen, Lilley, Laine, & Wahlberg, 2013; Vesterinen et al., 2016; Vesterinen, Puisto, & Blomberg, 2018; Wirta et al., 2015). Correspondingly, the available prey species (Lepidoptera) can be determined by collecting the feces of larvae (hereafter frass). To our knowledge for the first time in this context, we apply DNA metabarcoding to samples from both bird feces and insect frass to observe consumed prey and evaluate the food availability in the wild. Sampling of frass is already commonly used to determine caterpillar abundance in the canopy (e.g., Zandt, 1994; Rytkönen & Orell, 2001), as feces are produced in proportion to caterpillar biomass (Tinbergen & Dietz, 1994).

Firstly, we focused on whether we can extract, amplify and sequence the invertebrate DNA from both the bird and the insect feces. Secondly, we tested how well the sequences are identifiable on the grounds of the extensive regional DNA barcode reference library built by the national initiative, the Finnish Barcode of Life project (FinBOL, www.finbol.org), with the data maintained by BOLD Systems (Ratnasingham & Hebert, 2007). Thirdly, we investigated how much material is needed for accurate species identification from frass samples using a simple laboratory experiment with a single moth species. Finally, we analyzed whether varying amounts of DNA sequence material can be used to estimate the quantity of prey that is available and used by the predators. The success in this approach may open new perspectives in the studies of diet selection, food webs, and ecosystem functioning, including interspecific competition and resource partitioning.

## 2 METHODS

The study was carried out at field sites in Oulu (65°08ʹN, 25°53ʹE) and Kuusamo (66°02ʹN, 29°05ʹE), Finland. The fecal samples of most bird nestlings (willow tits *Poecile montanus*, great tits *Parus major* and blue tits *Cyanistes caeruleus*) and insects were collected at the Oulu site, except for those of Siberian tits (*Poecile cinctus*), which were collected in the study site in Kuusamo. These areas are typical Finnish mixed forest landscape with Scots pine *Pinus sylvestris*, Norwegian spruce *Picea abies* and birches *Betula *spp. as the dominant tree species (Rytkönen & Krams, 2003; Vatka, Orell, & Rytkönen, 2016). *Samia cynthia* (Lepidoptera, Saturniidae) caterpillars used for the laboratory experiment were reared in university facilities. Animal handling was done according to ethical guidelines presented by the National Animal Experiment Board.

### 2.1 Collection of nestling feces

The fecal samples of parid nestlings were collected in 2015 when the nestlings were about two weeks old (nearly three weeks for the Siberian tits), around mid‐June. All feces acquired at a nest (typically 1 to 3 fecal sacs) were collected in the same 5‐ml plastic tube containing 96% ethanol. The tubes were stored in a freezer at −20°C until analysis. The total number of fecal samples analyzed in this study was 14 (willow tits: 4, great tits: 4, blue tits: 2, Siberian tits: 4). This number was deemed adequate for a proof‐of‐concept study while simultaneously being manageable, and we tried to keep sampling effort even between the study species.

### 2.2 Frass collection in the wild

Frass of moth caterpillars was collected 1996–2014 using plastic funnels (Ø 35 cm) attached to tree trunks under the canopy. Only birches (*Betula* spp.) were selected for the frass collection because they are known to form the most important foraging habitat during the nestling period of the focal birds (Rytkönen & Krams, 2003). The frass was collected into paper coffee filters attached under the funnels. These samples were collected from the field site once a week. The frass samples were then either dried (1996–2011) or frozen at −20°C (2012 onwards) until analysis. The number of frass pellets in each sample was counted, and the diameter of five randomly chosen frass pellets was measured. This enables both the estimations for caterpillar biomass in the canopy (see Rytkönen & Orell, 2001) and of frass dry mass from the number of frass pellets. The frass analyzed in this study consists of 2 samples from 1996, 2 from 2004, 16 from 2012, and 4 from 2014. The samples were selected to match the annual caterpillar peak abundance (typically mid‐June). The average number of frass pellets per analyzed sample was 119 (range 15–320).

### 2.3 Laboratory experiment to test DNA barcoding with frass

To analyze how much frass is needed for successful species determination via metabarcoding, we used *Samia cynthia* (Lepidoptera, Saturniidae) as a model species. The larvae originated from a commercial laboratory stock. Eggs were housed at 20°C. Once the eggs hatched, larvae (*N* = 20) were divided evenly between two clean plastic containers provided with mesh lids to prevent the larvae from escaping. The larvae were reared at 20°C (light:dark cycle: 12 hr:12 hr) and fed ad libitum on fresh leaves of the cherry (*Prunus cerasus*, Rosaceae), which is a known host plant of the species. In the fifth larval instar, frass of the larvae was collected immediately after being observed in a container, and stored in a freezer at −20°C. Before analysis, two frass sets each produced by several individual caterpillars was dried, grinded, mixed, and divided into weight classes: 0.1, 0.3, 1.2, 4.2, 14.4, and 50 mg. Samples were weighed with a precision balance (Mettler Toledo MT 5).

### 2.4 DNA extraction of all samples

Before DNA extraction, the samples were unfrozen, homogenized, and dried at 50°C until the ethanol had vaporized. We used the QiaAmp Fast DNA Stool Mini Kit (ID: 51604, QIAGEN), which is specifically developed for fecal samples, for the extraction, and followed the manufacturer's instructions. The extracted DNA was further concentrated by evaporating samples in vacuum. A negative control treatment was carried out with each extraction batch, containing all the same chemicals but without any DNA sample. No products were formed in any of the extraction blanks, and they were not used in further sequencing analysis.

### 2.5 PCR and library construction

The DNA of fecal samples is generally highly fragmented (Deagle, Eveson, & Jarman, 2006), and therefore, short mini‐barcode primers (ZBJ‐ArtF1c/ZBJ‐ArtR2c) were selected for amplification (Zeale, Butlin, Barker, Lees, & Jones, 2011). These primers selectively amplify a 157 bp long target in mitochondrial *cytochrome c oxidase subunit I* (COI) gene in arthropods. Although the use of COI as a standard DNA barcode marker has received some critique for taxonomic bias (Clarke, Soubrier, Weyrich, & Cooper, 2014; Elbrecht & Leese, 2017), it has nevertheless been successfully applied in numerous studies and is by far the most commonly applied primers in studies targeting arthropod prey (e.g., Vesterinen et al., 2013, Clare et al., 2014, Wirta et al., 2015, Kaunisto et al., 2017). COI barcodes also have been used in one of the few dietary studies combining molecular data from both consumed arthropods and available arthropod prey (Vesterinen et al., 2016). Moreover, the complete COI reference library for ~2,600 species of Finnish Lepidoptera has proven its high functionality (Mutanen et al., 2016). Short sequences have previously been shown to be highly informative, allowing differentiation of species even in notoriously highly similar Lepidoptera (Hajibabaei et al., 2006; Mutanen, Kekkonen, Prosser, Hebert, & Kaila, 2015).

A two‐stage PCR process was used to amplify and prepare sequencing libraries (Carew, Pettigrove, Metzeling, & Hoffmann, 2013). In addition to the locus‐specific primers, linker sequences that allow for the easy inclusion of unique tags and adaptors can be attached in the second round (Clarke, Czechowski, Soubrier, Stevens, & Cooper, 2014). The first PCR step used the following primers:

ZBJ‐ArtF1c, 5ʹ‐CGCAGAGAGGCTCCGTG‐AGATATTGGAACWTTATATTTTATTTTTGG‐3ʹ, ZBJ‐ArtR2c, 5ʹ‐CAGGACCAGGGTACGGTG‐WACTAATCAATTWCCAAATCCTCC‐3ʹ.

The first step PCR was performed in 25 μl total volume including 12.5 μl Phusion^®^ Flash High‐Fidelity PCR Master Mix with GC Buffer (Thermofisher), 0.5 μM of forward and reverse primers, 2.5 μl of DNA template, and 7.5 μl of sterile water. PCR cycling profile was as follows: first denatured at 98°C for 2 min, followed by 38 cycles of 98°C for 10 s, 58°C for 30 s, and 72°C for 15 s, and then, after the last cycle hold at 72°C for 5 min. Two PCR reactions were prepared for each sample to offset PCR stochasticity and enhance detection of rare taxa (Alberdi, Aizpurua, Gilbert, & Bohmann, 2018), and combined after the PCR. Amplified PCR products were then purified with AMPure XP (Agencourt) at a 1.2× ratio, and concentration was measured using the MultiNA capillary electrophoresis system (Shimadzu).

In the second PCR stage, we used primers with unique 9‐base‐specific index‐tags to track each individual sample. Besides index‐tags, the primers had Ion Torrent PGM‐specific adaptors A and TrP1 as well as universal linkers (the same as first‐stage primers). The primer sequences used in the second step were (index‐tags marked with “N”):

AT1B‐XX, 5ʹ‐CCATCTCATCCCTGCGTGTCTCCGACTCAG‐NNNNNNNNN‐CAGGACCAGGGTACGGTG‐3ʹ,
TrP1T2, 5ʹ‐CCTCTCTTGGGCAGTCGGTGATC‐GCAGAGAGGCTCCGTG‐3ʹ.

The second PCR contained 25 μl Phusion^®^ High‐Fidelity Flash PCR Master Mix with GC Buffer (Thermofisher), 0.5 μM of forward and reverse primers, 10 ng of the purified PCR product from the previous step, and the total volume was brought to 50 μl with sterile water. Samples were initially denatured for 90 s at 98°C, followed by eight amplification cycles: 98°C for 10 s, 63°C for 20 s, and 72°C for 20 s, and after the cycles 72°C for 5 min.

The second PCR products were purified with AMPure XP at a 1.0× ratio and concentration was determined via picogreen dsDNA reagent (Thermofisher) after which the samples were pooled in equimolar ratios (25 ng of each sample). Pooled sample was further purified with AMPure XP at first in a 1.0× ratio, then double‐sided 0.6× and 1.2× ratios and finally at 1.0x ratio. Prior to sequencing, library profile was checked using the MultiNA capillary electrophoresis system and final concentration was determined with the picogreen reagent.

### 2.6 Sequencing of the pooled library

The sequencing of DNA was done at the Biocenter Oulu Sequencing Center, University of Oulu, by using Ion Torrent PGM device with a 316 chip. The manufacturer's instructions were followed and the following packages used: Ion PGM™ Template OT2 400 kit (Thermofisher) and Ion PGM™ Hi‐Q™ Sequencing Kit (Thermofisher).

### 2.7 Bioinformatics for the Ion Torrent data

Sequencing resulted in 3,484,589 raw reads of which about 1.8 million were COI amplicons. Resulting reads were processed with QIIME software (version 1.8.0, Caporaso et al., 2010). The raw FASTQ file was split on the basis of sample‐specific index‐tags into their own groups and low quality (average quality <Q20) raw reads were discarded (split libraries command). For passed reads, the presence of both forward and reverse primer sequence (from the first PCR stage) was required. Next, the reads were clustered based on their similarity (pick_otus de novo command) into Operational Taxonomic Units (OTUs). As sequences are particularly similar for the anticipated main prey (lepidopterans; Hebert et al., 2003), a low clustering value could result in clumping of closely related species together. Thus, we decided to use the 98% similarity threshold for clustering. For each OTU, a random sequence was selected as a representative sequence for the cluster (pick_rep_set command). Finally, an OTU table was made (make_otu_table command). OTUs with low abundance (<8 reads in all samples combined) were discarded from further analysis. Finally, 760 OTUs remained to be assigned to biological species.

### 2.8 Assignation of OTUs to species

We compared the OTUs to the international BOLD database (Ratnasingham & Hebert, 2007). Sequences were manually compared to the full BOLD database, which includes all the sequences uploaded into the BOLD systems, also those with no proper species assignation. The regional DNA barcode reference library is unusually comprehensive for insects, and for the most important prey group of tits, the Lepidoptera, its coverage is 100% (all species occurring in the area represented). The details of the assignation of the OTUs to species is in the supplements (Supplement 1: Assignation of OTUs to species).

The following basic principles were used in the determination of sequences:

1. 97%–100% match to the sequence in the database was required for the species determination, whereby the best match would be chosen as the species corresponding to the sample.
2. 97%–100% similarity to the database was also used as the threshold for genus determination.
3. If the species with the best match does not occur in the study area, a resident species with the next best matching sequence was selected instead, but only when the sequence showed >97% similarity to that species too.
4. The determination was left at the genus level (or another higher taxonomic level) if two species (or higher taxon) were equally probable on the basis of similarity of the sequences. The determination was also left at a higher taxonomic level if no sufficiently similar species (or higher taxon) could be found among the sequences in the database.
5. The determination was left to the order or family level if similarity was less than 97%.
6. Similarities below 90% were classified as unidentified to any taxonomic level. Often, the 20 best matches of such undefined sequences could belong to wholly different orders rendering class‐level determinations unreliable.
7. The identification was further left at a higher taxonomic level if more detailed determination was not available in the BOLD database.
8. Clear mistakes in the database were ignored, instead choosing the next best match.
9. All species from the control sample sequences were determined as the genus *Samia* and identified as *Samia *sp. (all should belong to the laboratory‐reared *Samia cynthia*). Other sequences in the control samples were classified as contamination.

Three OTUs were determined to species level at 95% identity, as a different OTU from the same sample had already been determined to the same species based on 100% identity. In a similar fashion, nine others were determined to the genus level. Finally, another four were determined to the genus level despite below 97% identity as the majority of their similar hits belonged to the same genus.

### 2.9 Statistical analyses

The R Statistical Environment (R Core Team, 2013) was used for the parametric tests and linear modeling on the relationships between sample size and metabarcoding success, as well as for the correlation analysis between prevalence and read abundance.

## 3 RESULTS

### 3.1 Success in DNA extraction and PCR

DNA extraction and PCR yielded sufficient DNA for further analysis in 31 of 50 samples (62%). This can be broken down to the following for the various categories of samples: 11/14 fecal samples of birds (79%), 8/24 frass samples of wild moth larvae (33%), and 12/12 frass samples of laboratory‐reared larvae (100%). The oldest successful frass samples were 20 years old (dating from 1996). The bird feces were collected during the preceding breeding season (2015), that is, about one year old. The success rate of DNA extractions from frass samples from the field sites seemed to be related to preservation method and the amount and quality of plant material in the samples. For dried frass, 4/4 attempts (100%) were successful, but for frozen frass samples, which included more plant fibers that were difficult to grind, this number decreased to 4/20 (20%).

### 3.2 Identification of DNA sequences

After filtering and trimming, the field samples (bird feces, frass samples) produced 760 OTUs of which 721 (95%) were identifiable to order level when compared to BOLD libraries. Correspondingly, 564 (74%) were identifiable to family level, 498 (66%) to genus level and 459 (60%) to species level. Of all species‐level identifications, 316 (69%) were done with a 100% match to the BOLD library sequences, 140 (31%) were done with match range 97%–100%, and 3 (<1%) with match range 94%–97%.

The list of all identified species, split between frass and parid samples, is presented in Supporting Information Appendix S1. The vast majority of identifications in both data sets belonged to the order Lepidoptera: 84% of identified sequences and 80% of species detected in bird feces, while this was respectively the case for 58% and 64% in the frass data.

### 3.3 The amount of fecal material and metabarcoding success

**Figure 1:**
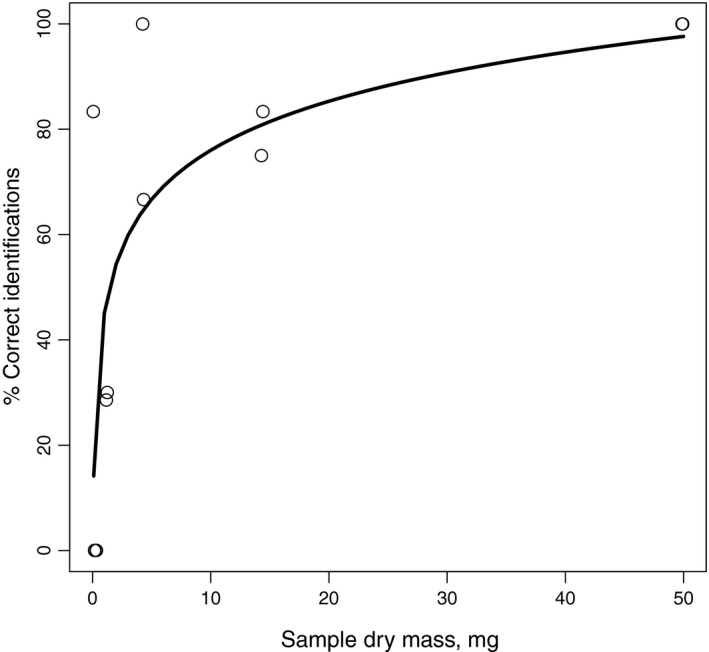
The relationship between frass sample dry mass (mg) and percentage of correct identifications in the feeding trial of lepidopteran species Samia cynthia

**Figure 2:**
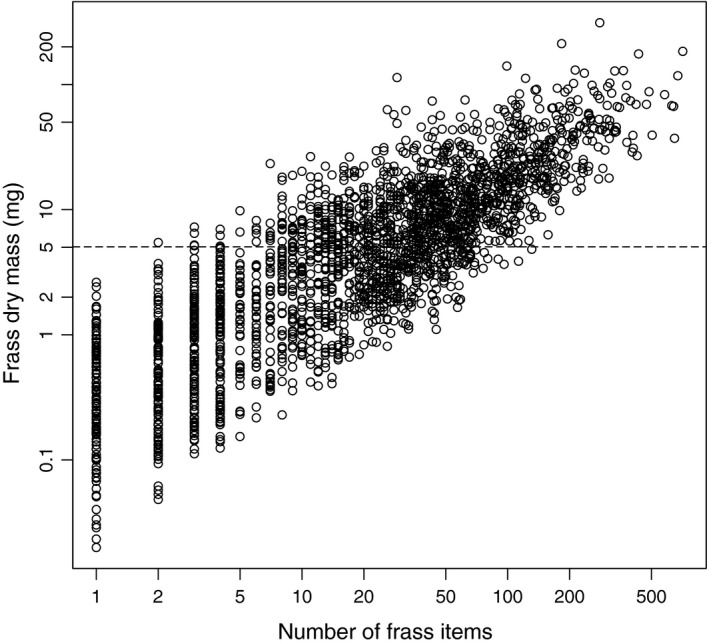
The relationship between frass dry mass (in mg) and number of frass pellets for material from the field site. The dashed reference line indicates the 5 mg frass mass which proved to be the threshold level for successful identification of a single moth species (Figure 1)

The mass of wild moth frass samples was not related to success in metabarcoding (i.e., successful and unsuccessful samples did not differ in their mass: *t* = −0.082, *df* = 8, *p* = 0.936). As these samples concern bulk material, however, no data of frass mass for individual moth species were available. The frass mass of the laboratory‐reared *Samia cynthia* was significantly related to success in metabarcoding. The best model included dry mass as log‐transformed variable (Table 1). The proportion of correct identifications increased sharply with increasing sample dry mass, especially when dry mass exceeded 5 mg (Figure 1). The corresponding frass mass in field collections could be achieved by collecting ca. 20 average‐sized frass pellets (per species); although the number cane range from 2 to 100 frass pellets due to substantial variation in their size (Figure 2).

**Table 1:** Specifications of the best linear model explaining the relationship between frass sample dry mass (mg) and the percentage of correct species identifications in the feeding trial (where all samples were known to be of *Samia cynthia*)

Explanatory variable	Estimate	*SE*	*t* Value	Pr(>|*t*|)
(Intercept)	45.075	9.038	4.987	0.000547
log(sample dry mass)	13.433	3.959	3.393	0.006855

Note

The adjusted *R*‐squared: 0.4886.

### 3.4 Quantitative data on species abundance

**Figure 3:**
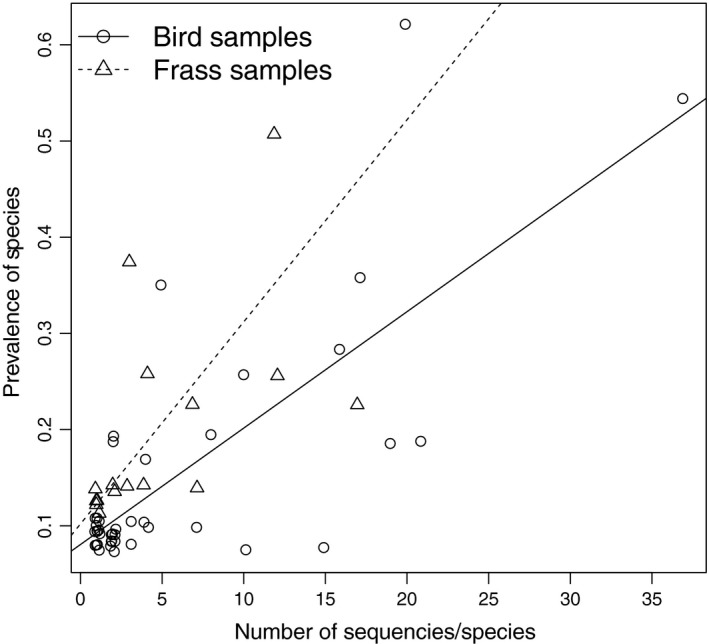
The relationship between the number of different identified sequences of a species within DNA samples and the prevalence of lepidopteran species in the whole data. Results for bird and frass samples separated. Data points represent individual lepidopteran species

We found a correlation between the number of different DNA sequences (in a sample) and the fraction of samples including a species (prevalence). The higher the number of sequences belonging to the same species, the more likely it is that these species are prevalent in our data, both in frass and bird samples (*r* = 0.807 and 0.755, *df* = 15 and 39 for frass and bird data, respectively, both *p* < 0.001). It should be noted, however, that this effect is mainly caused by the high variation in sequences for a few species, as can be seen in Figure 3.

## 4 DISCUSSION

Our study aimed to test the feasibility of conducting a combined research on the feces of both arthropod prey and their avian predators, in order to obtain insights into prey abundance and the corresponding predator diet. The dietary results we obtained match those in previous observations. Whether expressed as the proportion of species (77%), unique sequences (84%) or samples they occurred in (79%), the proportion of lepidopteran species in the birds’ feces is similar to the three quarters of prey items found for great and blue tits (Rytkönen & Krams, 2003) and the 20%–80% for willow tits (Rytkönen et al., 1996). By using metabarcoding combined with an expansive reference library, however, this large generalized group could be further investigated, and we were frequently able to identify prey accurately to the species level. The arthropod species encountered in the diet and environment contained those one would expect (e.g., moth species whose caterpillars feed on birch leaves), and the frass collection specifically also included nonlepidopterans (e.g., spiders, dipterans) accidentally trapped in the funnels. Although the latter could complicate quantitative comparisons across all species, their confirmed presence in the birds' habitat would still be of use in qualitative analyses. Only some species found in the nestlings' feces were also encountered in the frass samples and vice versa. Given the multitude of insect species occurring in the area, only a fraction was observed using either method, thus leading to a small overlap between the two. Our limited sample size is partially to blame for this, with most species only being found in one or two samples (see Supporting Information Appendix S1). The phenomenon of low overlap between available and detected prey is not uncommon even with larger sampling size however (Eitzinger et al., 2018; Vesterinen et al., 2016).

By experimentally manipulating the amount of fecal mass analyzed, we showed that the used metabarcoding method is powerful enough to detect DNA sequences from small amounts of material. However, a certain threshold level seems to exist, which may affect the detectability of scarce prey species in bulk samples. Based on the data from the laboratory‐raised moth species, the threshold to detect DNA with high probability was about 5 mg of dry frass mass. At present, we do not know if the problems with smaller samples already occur in the DNA extraction phase, during PCR, or whether some sequences are filtered from data due to their rarity. We also did not test if the amplification of the total genomic DNA might have a positive effect on this. Based on our long‐term frass data, the threshold value of 5 mg dry frass mass corresponds to ca. 20 average‐sized frass pellets, ranging from 2 to 100 depending on the frass pellet size (Figure 2). It is therefore recommended that frass samples used for metabarcoding analyses should be large where possible. On the other hand, having several separate frass samples may be used to obtain the quantitative prevalence data for the estimation of relative abundances of different moth species.

Our results show that invertebrate prey species can be identified from both predator feces and feces of the prey species. When DNA from predators' diets and their available prey species are representatively sampled, one could obtain detailed information not only on the diet of predators and the food web structure, but also on the predators’ preferred prey and niche differences between species. Existing knowledge on the ecology of parids (e.g., Rytkönen et al., 1996, Rytkönen & Krams, 2003) allowed us to focus on particular sections of their habitat: the birches, inhabited by moth caterpillars, which are the main foraging site during the nestling period. Metabarcoding analysis of these frass samples extends the traditional method of frass sampling to determine the overall abundance of caterpillars in the canopy (Tinbergen & Dietz, 1994) by allowing the detection of specific prey species. Moreover, although DNA metabarcoding is typically used for qualitative analyses of species, it may nonetheless be possible to acquire insights regarding quantitative data on species’ abundances as well (see Deagle et al., 2018). This can be done by utilizing intraspecific variation in the samples, indicating the occurrence of different individuals of the same species. The relationship we found between the number of different DNA sequences in a sample and the prevalence of a species seems to support this idea (Figure 3). This methodology could have promising applications in ecological studies of trophic interactions between insectivorous bird species and their invertebrate prey.

Given the limitations of our study, most notably the small sample size as well as the fact that bird and frass samples were collected in different years, it is too early to draw any sweeping conclusions on the ecological interactions within this food web. Indeed, the substantial variation in identified species in different samples indicates a requirement for a large number of samples, both for the bird feces and frass. Given fluctuations in arthropod numbers between the years, simultaneous collections of both types of samples would be preferred whenever possible, especially since our results suggest a broad range of arthropods to be predated upon by the birds. If already available, however, we have found that well‐stored historical samples can still yield good results using metabarcoding.

In this study, we demonstrated that metabarcoding can be employed to both determine a predator's diet from their feces (bird nestling feces) and the availability of those prey from their own feces (moth caterpillar frass), thus being applicable to trophic interaction studies. To our knowledge, this is the first time when the available insect prey species were successfully identified from prey feces using DNA metabarcoding. To validate our approach, we carried out a laboratory feeding experiment, and to prove the power of the method, we analyzed the diet of four widespread bird species. As both nestling feces and insect frass can be collected easily using harmless and unobtrusive methods, this methodology is ideal for many applications. With advances in sequencing technologies, detecting prey DNA from predator feces has recently become a norm in diet studies of many insectivorous animals (e.g., Clare et al., 2009; Wirta et al., 2015; Kaunisto et al., 2017; Vesterinen et al., 2018). Equipped with another new tool to dissect the diet of animals, we hope that our study opens a new wave of research utilizing both available and consumed prey.

## CONFLICT OF INTEREST

None declared.

## AUTHORS CONTRIBUTION

SR, EV, MH, and MO were primarily responsible for the collection of field samples (SR&EV great tit and blue tit, MH Siberian tit, MO willow tit). TL participated in the lab work. MS was responsible for the sequencing. PV, MM, and CW worked on the arthropod identification. EJV contributed to the final analyses. Writing and revision of the manuscript were primarily conducted by CW, EJV, SR, EV, and TL (methods), with contributions from the other authors.

## DATA ACCESSIBILITY

OUT sequences and OTU table are available in the Dryad Digital Repository: https://doi.org/10.5061/dryad.4f1n785.

## References

[ece34787-bib-0001] Alberdi, A. , Aizpurua, O. , Gilbert, M. T. P. , & Bohmann, K. (2018). Scrutinizing key steps for reliable metabarcoding of environmental samples. Methods in Ecology and Evolution, 9, 134–147. 10.1111/2041-210X.12849

[ece34787-bib-0004] Caporaso, J. G. , Kuczynski, J. , Stombaugh, J. , Bittinger, K. , Bushman, F. D. , Costello, E. K. , … Knight, R. (2010). QIIME allows analysis of high‐throughput community sequencing data. Nature Methods, 7, 335–336. 10.1038/nmeth.f.303 20383131 PMC3156573

[ece34787-bib-0005] Carew, M. E. , Pettigrove, V. J. , Metzeling, L. , & Hoffmann, A. A. (2013). Environmental monitoring using next generation sequencing: Rapid identification of macroinvertebrate bioindicator species. Frontiers in Zoology, 10, 45 10.1186/1742-9994-10-45 23919569 PMC3750358

[ece34787-bib-0006] Clare, E. L. , Fraser, E. E. , Braid, H. E. , Fenton, M. B. , & Hebert, P. D. N. (2009). Species on the menu of a generalist predator, the eastern red bat (*Lasiurus borealis*): Using a molecular approach to detect arthropod prey. Molecular Ecology, 18(11), 2532–2542. 19457192 10.1111/j.1365-294X.2009.04184.x

[ece34787-bib-0007] Clare, E. L. , Symondson, W. O. C. , Broders, H. , Fabianek, F. , Fraser, E. E. , MacKenzie, A. , … Reimer, J. P. (2014). The diet of *Myotis lucifugus* across Canada: Assessing foraging quality and diet variability. Molecular Ecology, 23(15), 3618–3632. 10.1111/mec.12542 24274182

[ece34787-bib-0008] Clarke, L. J. , Czechowski, P. , Soubrier, J. , Stevens, M. I. , & Cooper, A. (2014). Modular tagging of amplicons using a single PCR for high‐throughput sequencing. Molecular Ecology Resources, 14, 117–121. 24028345 10.1111/1755-0998.12162

[ece34787-bib-0009] Clarke, L. J. , Soubrier, J. , Weyrich, L. S. , & Cooper, A. (2014). Environmental metabarcodes for insects: In silico PCR reveals potential for taxonomic bias. Molecular Ecology Resources, 14(6), 1160–1170. 10.1111/1755-0998.12265 24751203

[ece34787-bib-0010] Cushing, D. H. (1969). The regularity of the spawning season of some fishes. ICES Journal of Marine Science, 33(1), 81–92. 10.1093/icesjms/33.1.81

[ece34787-bib-0011] Cushing, D. H. (1990). Plankton production and year‐class strength in fish populations: An update of the match/mismatch hypothesis. Advances in Marine Biology, 26, 249–293.

[ece34787-bib-0012] Deagle, B. E. , Eveson, J. P. , & Jarman, S. N. (2006). Quantification of damage in DNA recovered from highly degraded samples – a case study on DNA in faeces. Frontiers in Zoology, 3, e11. 10.1186/1742-9994-3-11 PMC1564134 16911807

[ece34787-bib-0013] Deagle, B. E. , Thomas, A. C. , McInnes, J. C. , Clarke, L. J. , Vesterinen, E. J. , Clare, E. L. , … Eveson, J. P. (2018). Counting with DNA in metabarcoding studies: How should we convert sequence reads to dietary data? Molecular Ecology, 1–16. 10.1111/mec.14734 29858539 PMC6905394

[ece34787-bib-0014] Eitzinger, B. , Abrego, N. , Gravel, D. , Huotari, T. , Vesterinen, E. J. , & Roslin, T. (2018). Assessing changes in arthropod predator‐prey interactions through DNA‐based gut content analysis – Variable environment, stable diet. Molecular Ecology, 1–15. 10.1111/mec.14872 30230073

[ece34787-bib-0015] Elbrecht, V. , & Leese, F. (2017). Validation and development of COI metabarcoding primers for freshwater macroinvertebrate bioassessment. Frontiers in Environmental Science, 5, 11 10.3389/fenvs.2017.00011

[ece34787-bib-0016] Gibson, J. , Shokralla, S. , Porter, T. M. , King, I. , van Konynenburg, S. , Janzen, D. H. , … Hajibabaei, M. (2014). Simultaneous assessment of the macrobiome and microbiome in a bulk sample of tropical arthropods. Proceedings of the National Academy of Sciences of the United States of America, 111, 8007–8012. 24808136 10.1073/pnas.1406468111 PMC4050544

[ece34787-bib-0017] Hajibabaei, M. , Smith, M. , Janzen, D. , Rodriguez, J. , Whitfield, J. , & Hebert, P. D. N. (2006). A minimalist barcode can identify a specimen whose DNA is degraded. Molecular Ecology Notes, 6, 959–964. 10.1111/j.1471-8286.2006.01470.x

[ece34787-bib-0018] Hebert, P. D. N. , Ratnasingham, S. , & deWaard, J. R. (2003). Barcoding animal life: cytochrome c oxidase subunit 1 divergence among closely related species. Proceedings of the Royal Society of London B: Biological Sciences, 270, S96–S99. 10.1098/rsbl.2003.0025 PMC1698023 12952648

[ece34787-bib-0019] Jedlicka, J. A. , Sharma, A. M. , & Almeida, R. P. P. (2012). Molecular tools reveal diets of insectivorous birds from predator fecal matter. Conservation Genetics Resources, 5(3), 879–885. 10.1007/s12686-013-9900-1

[ece34787-bib-0020] Kaunisto, K. M. , Roslin, T. , Sääksjärvi, I. E. , & Vesterinen, E. J. (2017). Pellets of proof: First glimpse of the dietary composition of adult odonates as revealed by metabarcoding of feces. Ecology and Evolution, 7(20), 8588–8598. 10.1002/ece3.3404 29075474 PMC5648679

[ece34787-bib-0021] King, R. A. , Read, D. S. , Traugott, M. , & Symondson, W. O. C. (2008). Molecular analysis of predation: A review of best practice for DNA‐based approaches. Molecular Ecology, 17, 947–963. 18208490 10.1111/j.1365-294X.2007.03613.x

[ece34787-bib-0022] Meusnier, I. , Singer, G. A. C. , Landry, J.‐F. , Hickey, D. A. , Hebert, P. D. N. , & Hajibabaei, M. (2008). A universal DNA mini‐barcode for biodiversity analysis. BMC Genomics, 9, 214 10.1186/1471-2164-9-214 18474098 PMC2396642

[ece34787-bib-0023] Moreby, S. J. , & Stoate, C. (2000). A quantitative comparison of neck‐collar and faecal analysis to determine passerine nestling diet. Bird Study, 47, 320–331. 10.1080/00063650009461192

[ece34787-bib-0024] Mutanen, M. , Kekkonen, M. , Prosser, S. W. , Hebert, P. D. , & Kaila, L. (2015). One species in eight: DNA barcodes from type specimens resolve a taxonomic quagmire. Molecular Ecology Resources, 15(4), 967–984. 10.1111/1755-0998.12361 25524367 PMC4964951

[ece34787-bib-0025] Mutanen, M. , Kivelä, S. M. , Vos, R. A. , Doorenweerd, C. , Ratnasingham, S. , Hausmann, A. , … Godfray, H. C. J. (2016). Species‐level para‐ and Polyphyly in DNA barcode gene trees: strong operational bias in European Lepidoptera. Systematic Biology, 65, 1024–1040. 10.1093/sysbio/syw044 27288478 PMC5066064

[ece34787-bib-0026] Naef‐Daenzer, L. , Naef‐Daenzer, B. , & Nager, R. G. (2000). Prey selection and foraging performance of breeding Great Tits *Parus major* in relation to food availability. Journal of Avian Biology, 31, 206–214. 10.1034/j.1600-048X.2000.310212.x

[ece34787-bib-0027] Paine, R. T. (1966). Food web complexity and species diversity. The American Naturalist, 100, 65–75. 10.1086/282400

[ece34787-bib-0028] Petchey, O. L. , Beckerman, A. P. , Riede, J. O. , & Warren, P. H. (2008). Size, foraging, and food web structure. Proceedings of the National Academy of Sciences of the United States of America, 105, 4191–4196. 10.1073/pnas.0710672105 18337512 PMC2393804

[ece34787-bib-0029] Pompanon, F. , Deagle, B. E. , Symondson, W. O. C. , Brown, D. S. , Jarman, S. N. , & Taberlet, P. (2012). Who is eating what: Diet assessment using next generation sequencing. Molecular Ecology, 21(8), 1931–1950. 10.1111/j.1365-294X.2011.05403.x 22171763

[ece34787-bib-0031] R Core Team (2013). R: A language and environment for statistical computing. Vienna, Austria: R Foundation for Statistical Computing Retrieved from http://www.R-project.org/

[ece34787-bib-0032] Ratnasingham, S. , & Hebert, P. D. N. (2007). BOLD: The barcode of life data system. Molecular Ecology Resources, 7(3), 355–364. 10.1111/j.1471-8286.2007.01678.x PMC1890991 18784790

[ece34787-bib-0034] Rytkönen, S. , Koivula, K. , & Orell, M. (1996). Patterns of per‐brood and per‐offspring provisioning in the willow tit *Parus montanus* . Journal of Avian Biology, 27, 21–30.

[ece34787-bib-0035] Rytkönen, S. , & Krams, I. (2003). Does foraging behaviour explain the poor breeding success of great tits *Parus major* in northern Europe? Journal of Avian Biology, 34, 288–297.

[ece34787-bib-0036] Rytkönen, S. , & Orell, M. (2001). Great tits (*Parus major*) lay too many eggs: Experimental evidence in mid‐boreal habitats. Oikos, 93, 439–450. 10.1034/j.1600-0706.2001.930309.x

[ece34787-bib-0037] Symondson, W. O. C. (2002). Molecular identification of prey in predator diets. Molecular Ecology, 11(4), 627–641. 10.1046/j.1365-294X.2002.01471.x 11972753

[ece34787-bib-0038] Tinbergen, J. M. , & Dietz, M. W. (1994). Parental energy expenditure during brood rearing in the great tit (*Parus major*) in relation to body mass, temperature, food availability and clutch size. Functional Ecology, 8, 563–572. 10.2307/2389916

[ece34787-bib-0039] Tiusanen, M. , Hebert, P. D. N. , Schmidt, N. M. , & Roslin , T. (2016). One fly to rule them all – Muscid flies are the key pollinators in the Arctic. Proceedings of the Royal Society B: Biological Sciences, 283(1839), 20161271 10.1098/rspb.2016.1271 PMC5046896 27683367

[ece34787-bib-0040] Vatka, E. , Orell, M. , & Rytkönen, S. (2016). The relevance of food peak architecture in trophic interactions. Global Change Biology, 22, 1585–1594. 10.1111/gcb.13144 26527602

[ece34787-bib-0041] Vesterinen, E. J. , Lilley, T. , Laine, V. N. , & Wahlberg, N. (2013). Next generation sequencing of fecal DNA reveals the dietary diversity of the widespread insectivorous predator Daubenton's bat (*Myotis daubentonii*) in Southwestern Finland. PLoS ONE, 8(11), e82168 10.1371/journal.pone.0082168 24312405 PMC3842304

[ece34787-bib-0042] Vesterinen, E. J. , Puisto, A. I. E. , Blomberg, A. , & Lilley , T. M. (2018). Table for five, please: Dietary partitioning in boreal bats. Ecology and Evolution, 1–24. 10.1002/ece3.4559 PMC6262732 30519417

[ece34787-bib-0043] Vesterinen, E. J. , Ruokolainen, L. , Wahlberg, N. , Peña, C. , Roslin, T. , Laine, V. N. , … Lilley, T. M. (2016). What you need is what you eat? Prey selection by the bat *Myotis daubentonii* . Molecular Ecology, 25(7), 1581–1594. 10.1111/mec.13564 26841188

[ece34787-bib-0044] Visser, M. E. , Van Noordwijk, A. J. , Tinbergen, J. M. , & Lessells, C. M. (1998). Warmer springs lead to mistimed reproduction in great tits (*Parus major*). Proceedings of the Royal Society of London B: Biological Sciences, 265, 1867–1870. 10.1098/rspb.1998.0514

[ece34787-bib-0045] Wirta, H. K. , Vesterinen, E. J. , Hambäck, P. A. , Weingartner, E. , Rasmussen, C. , Reneerkens, J. , … Roslin, T. (2015). Exposing the structure of an Arctic food web. Ecology and Evolution, 5(17), 3842–3856. 10.1002/ece3.1647 26380710 PMC4567885

[ece34787-bib-0046] Yu, D. W. , Ji, Y. , Emerson, B. C. , Wang, X. , Ye, C. , Yang, C. , & Ding, Z. (2012). Biodiversity soup: Metabarcoding of arthropods for rapid biodiversity assessment and biomonitoring. Methods in Ecology and Evolution, 3, 613–623. 10.1111/j.2041-210X.2012.00198.x

[ece34787-bib-0047] Zaidi, R. , Jaal, Z. , Hawkes, N. , Hemingway, J. , & Symondson, W. (1999). Can multiple‐copy sequences of prey DNA be detected amongst the gut contents of invertebrate predators? Molecular Ecology, 8(12), 2081–2087. 10.1046/j.1365-294x.1999.00823.x 10632859

[ece34787-bib-0048] Zandt, H. S. (1994). A comparison of three sampling techniques to estimate the population size of caterpillars in trees. Oecologia, 97, 399–406. 10.1007/BF00317331 28313636

[ece34787-bib-0049] Zeale, M. R. K. , Butlin, R. K. , Barker, G. L. A. , Lees, D. C. , & Jones, G. (2011). Taxon‐specific PCR for DNA barcoding arthropod prey in bat faeces. Molecular Ecology Resources, 11, 236–244. 21429129 10.1111/j.1755-0998.2010.02920.x

