# Supplementary material for: From feces to data: A metabarcoding method for analyzing consumed and available prey in a bird‐insect food web
Source: Ecol Evol. 2018 Dec 21;9(1):631–9. doi: 10.1002/ece3.4787 (PMC6342092; doi:10.1002/ece3.4787)
Supplement: Supplementary file 1 [file ECE3-9-631-s001.docx]

Appendix S1. The invertebrate species identified from the frass and parid data. The ‘sequences’ column lists the number of different sequences identified to that particular species, while ‘samples’ gives the number of samples containing that species.

|  |  |  |  | Sequences | | Samples | |
| --- | --- | --- | --- | --- | --- | --- | --- |
| Order | Family | Genus | Species | Frass | Parids | Frass | Parids |
| Araneae | Araneidae | Araneus | *Araneus marmoreus* | 0 | 4 | 0 | 2 |
| Araneae | Araneidae | Nuctenea | *Nuctenea silvicultrix* | 0 | 1 | 0 | 1 |
| Araneae | Philodromidae | Philodromus | *Philodromus cespitum* | 0 | 6 | 0 | 1 |
| Araneae | Theridiidae | Ohlertidion | *Ohlertidion ohlerti* | 1 | 15 | 1 | 2 |
| Araneae | Thomisidae | Xysticus | *Xysticus luctuosus* | 1 | 0 | 1 | 0 |
| Coleoptera | Curculionidae | Strophosoma | *Strophosoma capitatum* | 0 | 1 | 0 | 1 |
| Diptera | Ceratopogonidae | Culicoides | *Culicoides albicans* | 2 | 0 | 1 | 0 |
| Diptera | Chironomidae | Parakiefferiella | *Parakiefferiella coronata* | 1 | 0 | 1 | 0 |
| Diptera | Culicidae | Aedes | *Aedes communis* | 9 | 4 | 1 | 4 |
| Diptera | Culicidae | Culex | *Culex pipiens* | 0 | 1 | 0 | 1 |
| Diptera | Dolichopodidae | Dolichopus | *Dolichopus lepidus* | 1 | 0 | 1 | 0 |
| Diptera | Empididae | Rhamphomyia | *Rhamphomyia anomalipennis* | 22 | 0 | 1 | 0 |
| Diptera | Hybotidae | Euthyneura | *Euthyneura myrtilli* | 1 | 0 | 1 | 0 |
| Diptera | Muscidae | Helina | *Helina annosa* | 0 | 1 | 0 | 1 |
| Diptera | Muscidae | Spilogona | *Spilogona dispar* | 0 | 0 | 0 | 0 |
| Diptera | Psychodidae | Psychoda | *Psychoda gemina* | 1 | 0 | 1 | 0 |
| Diptera | Sciaridae | Corynoptera | *Corynoptera subdentata* | 1 | 0 | 1 | 0 |
| Diptera | Syrphidae | Syrphus | *Syrphus ribesii* | 0 | 3 | 0 | 1 |
| Diptera | Tachinidae | Dinera | *Dinera ferina* | 0 | 2 | 0 | 1 |
| Diptera | Tachinidae | Trixa | *Trixa caerulescens* | 0 | 1 | 0 | 1 |
| Diptera | Xylophagidae | Xylophagus | *Xylophagus ater* | 18 | 7 | 1 | 2 |
| Hemiptera | Adelgidae | Pineus | *Pineus pini* | 1 | 0 | 1 | 0 |
| Hemiptera | Aphididae | Cinara | *Cinara pruinosa* | 4 | 0 | 1 | 0 |
| Hemiptera | Aphididae | Euceraphis | *Euceraphis betulae* | 1 | 0 | 1 | 0 |
| Hymenoptera | Cimbicidae | Trichiosoma | *Trichiosoma lucorum* | 2 | 0 | 1 | 0 |
| Lepidoptera | Argyresthiidae | Argyresthia | *Argyresthia retinella* | 2 | 0 | 1 | 0 |
| Lepidoptera | Coleophoridae | Coleophora | *Coleophora serratella* | 0 | 2 | 0 | 1 |
| Lepidoptera | Depressariidae | Exaeretia | *Exaeretia ciniflonella* | 1 | 0 | 1 | 0 |
| Lepidoptera | Endromidae | Endromis | *Endromis versicolora* | 0 | 1 | 0 | 1 |
| Lepidoptera | Erebidae | Orgyia | *Orgyia antiqua* | 0 | 15 | 0 | 1 |
| Lepidoptera | Erebidae | Scoliopteryx | *Scoliopteryx libatrix* | 3 | 17 | 3 | 4 |
| Lepidoptera | Eriocraniidae | Eriocrania | *Eriocrania cicatricella* | 0 | 1 | 0 | 1 |
| Lepidoptera | Eriocraniidae | Eriocrania | *Eriocrania sangii* | 0 | 8 | 0 | 2 |
| Lepidoptera | Eriocraniidae | Eriocrania | *Eriocrania semipurpurella* | 0 | 21 | 0 | 2 |
| Lepidoptera | Eriocraniidae | Heringocrania | *Heringocrania unimaculella* | 0 | 1 | 0 | 1 |
| Lepidoptera | Geometridae | Alcis | *Alcis repandata* | 0 | 10 | 0 | 1 |
| Lepidoptera | Geometridae | Arichanna | *Arichanna melanaria* | 0 | 10 | 0 | 3 |
| Lepidoptera | Geometridae | Cleora | *Cleora cinctaria* | 0 | 1 | 0 | 1 |
| Lepidoptera | Geometridae | Crocallis | *Crocallis elinguaria* | 0 | 1 | 0 | 1 |
| Lepidoptera | Geometridae | Ematurga | *Ematurga atomaria* | 4 | 0 | 1 | 0 |
| Lepidoptera | Geometridae | Epirrita | *Epirrita autumnata* | 3 | 0 | 1 | 0 |
| Lepidoptera | Geometridae | Geometra | *Geometra papilionaria* | 0 | 1 | 0 | 1 |
| Lepidoptera | Geometridae | Idaea | *Idaea aversata* | 1 | 7 | 1 | 1 |
| Lepidoptera | Geometridae | Lycia | *Lycia hirtaria* | 0 | 1 | 0 | 1 |
| Lepidoptera | Geometridae | Operophtera | *Operophtera brumata* | 0 | 2 | 0 | 1 |
| Lepidoptera | Geometridae | Pasiphila | *Pasiphila debiliata* | 0 | 3 | 0 | 1 |
| Lepidoptera | Geometridae | Rheumaptera | *Rheumaptera hastata* | 12 | 0 | 2 | 0 |
| Lepidoptera | Hepialidae | Pharmacis | *Pharmacis fusconebulosa* | 1 | 16 | 1 | 3 |
| Lepidoptera | Hepialidae | Phymatopus | *Phymatopus hecta* | 1 | 0 | 1 | 0 |
| Lepidoptera | Lasiocampidae | Dendrolimus | *Dendrolimus pini* | 0 | 4 | 0 | 1 |
| Lepidoptera | Lasiocampidae | Poecilocampa | *Poecilocampa populi* | 0 | 2 | 0 | 2 |
| Lepidoptera | Noctuidae | Anaplectoides | *Anaplectoides prasina* | 12 | 20 | 4 | 7 |
| Lepidoptera | Noctuidae | Apamea | *Apamea remissa* | 0 | 2 | 0 | 1 |
| Lepidoptera | Noctuidae | Brachylomia | *Brachylomia viminalis* | 0 | 2 | 0 | 1 |
| Lepidoptera | Noctuidae | Colocasia | *Colocasia coryli* | 0 | 2 | 0 | 2 |
| Lepidoptera | Noctuidae | Eurois | *Eurois occulta* | 0 | 4 | 0 | 2 |
| Lepidoptera | Noctuidae | Graphiphora | *Graphiphora augur* | 17 | 5 | 2 | 4 |
| Lepidoptera | Noctuidae | Mniotype | *Mniotype adusta* | 1 | 0 | 1 | 0 |
| Lepidoptera | Noctuidae | Orthosia | *Orthosia incerta* | 0 | 1 | 0 | 1 |
| Lepidoptera | Noctuidae | Panolis | *Panolis flammea* | 0 | 1 | 0 | 1 |
| Lepidoptera | Noctuidae | Syngrapha | *Syngrapha interrogationis* | 0 | 19 | 0 | 2 |
| Lepidoptera | Noctuidae | Xestia | *Xestia baja* | 0 | 4 | 0 | 1 |
| Lepidoptera | Notodontidae | Notodonta | *Notodonta dromedarius* | 0 | 2 | 0 | 1 |
| Lepidoptera | Notodontidae | Pheosia | *Pheosia gnoma* | 24 | 37 | 6 | 6 |
| Lepidoptera | Nymphalidae | Aphantopus | *Aphantopus hyperantus* | 0 | 2 | 0 | 1 |
| Lepidoptera | Pyralidae | Ortholepis | *Ortholepis betulae* | 0 | 1 | 0 | 1 |
| Lepidoptera | Tortricidae | Adoxophyes | *Adoxophyes orana* | 2 | 0 | 1 | 0 |
| Lepidoptera | Tortricidae | Apotomis | *Apotomis turbidana* | 0 | 2 | 0 | 1 |
| Lepidoptera | Tortricidae | Epinotia | *Epinotia cinereana* | 7 | 0 | 1 | 0 |
| Lepidoptera | Tortricidae | Epinotia | *Epinotia maculana* | 7 | 1 | 2 | 1 |
| Lepidoptera | Tortricidae | Orthotaenia | *Orthotaenia undulana* | 0 | 2 | 0 | 1 |
| Lepidoptera | Tortricidae | Pandemis | *Pandemis cerasana* | 0 | 1 | 0 | 1 |
| Lepidoptera | Tortricidae | Phiaris | *Phiaris bipunctana* | 0 | 1 | 0 | 1 |
| Lepidoptera | Tortricidae | Rhyacionia | *Rhyacionia pinicolana* | 0 | 3 | 0 | 1 |
| Lepidoptera | Ypsolophidae | Ypsolopha | *Ypsolopha parenthesella* | 4 | 2 | 2 | 1 |
| Psocodea | Caeciliusidae | Valenzuela | *Valenzuela flavidus* | 1 | 0 | 1 | 0 |
| Psocodea | Peripsocidae | Peripsocus | *Peripsocus subfasciatus* | 6 | 0 | 1 | 0 |
